# USP14 predicts poorer survival outcomes and promotes tumor progression in endometrial carcinoma by activating NF-κB signaling

**DOI:** 10.18632/aging.205168

**Published:** 2023-11-01

**Authors:** Xiaojin Gong, Li Jia, Lili Zhou, Tongxiu Hu

**Affiliations:** 1 Department of Obstetrics and Gynecology, Tianjin Hospital, Tianjin 300211, China; 2 Department of Gynecology, Chongqing Traditional Chinese Medicine Hospital, Chongqing 400021, China; 3 Department of Nursing, Hejiang People’s Hospital, Luzhou, Sichuan 646200, China

**Keywords:** USP14, NF-κB, epithelial-mesenchymal transition, metastasis, endometrial carcinoma

## Abstract

Ubiquitin-specific protease 14 (USP14), a member of the USP family, which catalyzes ubiquitin cleavage from a range of protein substrates, has been found dysregulated in several cancers. Our aim is to explore the functions and mechanism of USP14 in endometrial carcinoma (EC). Quantitative real-time PCR (qRT-PCR) and western blot (WB) were used to assess USP14 levels in EC tissues and adjacent nontumor tissues. USP14 overexpression or knockdown models were adopted to determine USP14-mediated effects on EC cell proliferation, migration, invasion, apoptosis, and epithelial-mesenchymal transition (EMT). The xenograft tumor experiment checked the effect of USP14 overexpression on tumor cell growth. Furthermore, the upstream signaling pathway of USP14 was predicted by bioinformatics. Consequently, EC tissues exhibited USP14 overexpression compared to normal paracancerous nontumor tissues. USP14 presence was linked to an adverse prognosis in EC cases. Functionally, USP14 overexpression reduced apoptosis and increased cell migration, invasion, and EMT *in vivo* and *ex vivo*. USP14 knockdown had the opposite effect. Mechanistically, NF-κB pathway activation occurred through the inhibitory effect of USP14 on I-κB expression. Conversely, NF-κB pathway inhibition attenuated USP14-mediated carcinogenic effects. Additionally, there existed a binding interaction between miR-124-3p and the 3′-UTR of USP14, resulting in USP14 activity inhibition. In summary, our research indicates that the involvement of miR-124-3p in USP14 regulation contributes to exacerbated EC progression through NF-κB pathway activation. The modulation of this pathway may be a new strategy for treating EC.

## INTRODUCTION

Endometrial carcinoma (EC) ranks high among the prevalent malignancies affecting the female population. As a result of population aging and lifestyle changes, its incidence is gradually increasing [1, 2]. The established therapeutic approach for EC involves the implementation of total hysterectomy and bilateral salpingo-oophorectomy, either independently or in conjunction with pelvic and para-aortic lymph node dissection [3]. However, the recurrence rate of advanced EC remains high due to high metastasis [4]. Therefore, understanding the underlying mechanisms of EC development may contribute to developing more effective EC treatment strategies.

Nuclear factor-κB (NF-κB) belongs to a transcription factor family and plays a crucial role in the modulation of diverse biological responses, especially inflammation [5]. A growing body of evidence indicates that NF-κB has an essential role in the progression of tumors [6]. Notably, multiple studies have demonstrated that NF-κB activation is implicated in EC evolution [7]. For example, the glucose transporter GLUT6 is altered in EC samples and has a positive co-expression with NF-κB signalling pathway members. The RelA subunit of NF-κB pathway overexpression as well as TNFα treatment both promoted GLUT6 gene expression [8]. Thus, NF-κB is a vital mediator and therapeutic target in EC.

Deubiquitinase (DUB) is a major protease that regulates protein degradation. Ubiquitin-specific protease 14 (USP14) is a deubiquitinating enzyme that exerts a reverse regulatory effect on the activity of proteasomes [9]. Increasing studies have revealed that USP14 mediates inflammatory disease by activating NF-κB. For example, USP14 activates NF-κB by inducing IκBα deubiquitination and degradation, which enhances the dedifferentiation of IL-1β on chondrocytes in osteoarthritis [10]. Interestingly, accumulated studies have suggested that USP14 acts a pivotal protein in mediating cancer progression by affecting tumor cell proliferation, migration and angiogenesis [11]. For instance, hsa-miR-124a has downregulated level in non-small cell lung cancer (NSCLC) cells and tissues. USP14 is a direct target of miR-124a. USP14 downregulation by miR-124a led to reduced cell growth, stemness and enhanced gefitinib sensitivity [12]. Nonetheless, the precise role of USP14 in EC requires further investigation.

In this research, it was discerned that the USP14 level was elevated in EC and linked to the worse prognosis of EC. In addition, USP14 overexpression exerted an oncogenic role in EC and contributed to NF-κB activation. Furthermore, bioinformatics analysis demonstrated that miR-124-3p was an underlying target gene of USP14. Therefore, we posited that the miR-124-3p-USP14 axis potentially modulates EC development by modulating NF-κB pathway. To summarize, our evaluation of the biological significance and underlying mechanisms of USP14 in EC aims to uncover novel therapeutic targets for early diagnosis and advanced treatment of EC.

## MATERIALS AND METHODS

### Patients and tissues

A total of forty-four EC tissues and corresponding healthy tissues were included in this study. These samples were procured from EC patients who underwent surgical resection at Tianjin Hospital between January 2014 and March 2015. Prior to participation, all patients provided informed consent, and the study received approval from Tianjin Hospital. Following tumor removal, all tissues were promptly collected and preserved in liquid nitrogen at a temperature of −80°C.

### Culture and transfection of cells

The HEC-1-B and SNG-M human EC cell lines were procured from the Cell Center of the Chinese Academy of Sciences located in Shanghai, China. These cell lines were cultured in an incubator set at a temperature of 37°C with 5% CO_2_, utilizing RPMI1640 medium supplemented with 10% fetal bovine serum (FBS) and 1% penicillin/streptomycin obtained from Invitrogen (Carlsbad, CA, USA). Thermo Fisher Scientific, Inc. (Waltham, MA, USA) provided the RPMI1640 medium and FBS. Subculture of the cell lines during the logarithmic growth phase was performed using 0.25% trypsin sourced from Thermo Fisher HyClone (Logan, UT, USA). The pcDNA empty vector (referred to as NC), pcDNA-USP14 (referred to as USP14), shRNA normal control (referred to as sh-NC), shRNAs against USP14 (referred to as sh-USP14), miR-124-3p mimics, and the negative control miR-NC were all provided by GenePharma Co., Ltd. based in Shanghai, China. To facilitate transfection, Lipofectamine^®^ 3000 (Invitrogen; Thermo Fisher Scientific, Inc., Waltham, MA, USA) was utilized to deliver those vectors into Ishikawa cells, which had been cultured for 24 hours. The effectiveness of transfection was evaluated using qRT-PCR. Subsequently, the cells were incubated for a period of 24 hours at a temperature of 37°C under 5% CO_2_.

### Colony formation experiment

The HEC-1-B and SNG-M cells that underwent transfection were seeded into 6-well plates at a density of 1000 cells per well. Subsequently, these cells were cultured in RPMI-1640 medium supplemented with 10% FBS and 1% penicillin/streptomycin for a duration of two weeks. Following the incubation period, the cells were fixed using formaldehyde and stained with a 0.5% crystal violet solution. 10 min later, the staining was stopped. After being rinsed with PBS buffer three times, the colonies stained by crystal violet were visualized and counted under an optical microscope. This experimental procedure was repeated three times for each test.

### Flow cytometry (FCM)

Subsequent to the application of various treatments, HEC-1-B and SNG-M cells were subjected to trypsinization and subsequently harvested through centrifugation at a speed of 1500 rpm for 3 minutes. The resulting cells were then processed using the Annexin V-FITC/PI Apoptosis Detection Kit (Cat. No. 40302ES20, Yeasen, Shanghai, China) in the following manner. The cells were subjected to two rounds of washing with PBS, after which 400 μL of precooled PBS was added. Subsequently, 10 μL of Annexin V-FITC and 5 μL of PI were sequentially supplemented. The treated cells were incubated at a temperature of 4°C for a period of 30 minutes in darkness to facilitate apoptosis detection. FCM (cytoFLEX LX, Beckman Coulter, Indianapolis, IN, USA) was promptly employed to verify the occurrence of apoptosis. Computer software (Tree Star, Ashland, OR, USA) was utilized to calculate the percentage of apoptotic cells.

### Transwell assay

To assess cell migration and invasion, a Transwell assay was employed. Ishikawa cells were treated with 0.25% trypsin, followed by centrifugation, resuspension, and dispersion into 24-well plates. For the invasion experiment, 8-μm pore size chambers (Corning, Beijing, China) coated by Matrigel (BD354277, BD, Franklin Lake, NJ, USA) were utilized, whereas they were not utilized for the migration experiment. A total of 5 × 10^4^ transfected cells were placed in the upper chamber, and Matrigel was added. In contrast, the lower chamber was supplemented with 10% FBS medium, and 400 μL of RPMI-1640 was added. After incubation at 37°C for 24 hours, the uninvaded cells were carefully removed. The Transwell membranes were fixed using 4% paraformaldehyde (PFA) (P0099, Beyotime, Shanghai, China) for 10 minutes and subsequently stained with a 0.5% crystal violet solution. The membranes were then rinsed with tap water to remove excess stains, and the cells were counted. All experiments were performed three times.

### qRT-PCR

The extraction of total RNA was carried out using TRIzol reagent (Cat. No. 15596026, Invitrogen, Waltham, MA, USA). The concentration and purity of the RNA were determined using a Nanodrop spectrophotometer. Simultaneously, complementary DNA (cDNA) was synthesized from 1 μg of total RNA utilizing the PrimeScript-RT Kit from Promega (Madison, WI, USA). Subsequently, qRT-PCR was performed using SYBR^®^ Premix-Ex-Taq^™^ and the ABI7300 system obtained from Takara in TX, USA. The PCR system volume was set at 30 μL, and each sample contained 300 ng of cDNA. The amplification process commenced with an initial denaturation step at 95°C for 10 minutes, followed by 45 cycles at 95°C for 10 seconds, 60°C for 30 seconds, and 85°C for 20 seconds. The fluorescence data obtained were subjected to relative quantification, with GAPDH serving as the endogenous control for USP14 and U6 serving as the endogenous control for miR-124-3p. The qRT-PCR procedure was repeated three times. The primer sequences include: miR-124-3p, forward, 5′-CTCAACTGGTGTCGTGGAGTCGGCAATTCAGTTGAGGGCATTCA-3′; reverse, 5′-ACACTCCAGCT GGGTAAGGCACGCGGTGAATGCC-3′; USP14: forward, 5′-GTTGGAGCTTGGCTGAAGAC-3′, reverse, 5′-CTGCATTCAGCATCCAAAGA-3′; U6 forward, 5′-CTCGCTTCGGCAGCACA-3′, reverse, 5′-AACGCTTCACGAATTTGCGT-3′; GAPDH: forward, 5’-GATATTGTTGCCATCAATGAC-3′, reverse, 5′-TTGATTTTGGAGGGATCTCG-3′.

### Western blot (WB)

Following the treatment of the endometrial tissues and cells, the medium was removed and discarded. Subsequently, protein lysates obtained from Roche were added, facilitating the separation of total protein. 50 μg total protein from each group was then loaded onto a 12% polyacrylamide gel for electrophoresis at 100 V for 2 hours. The protein was subsequently transferred to polyvinylidene fluoride (PVDF) membranes. The membranes were blocked with 5% skimmed milk at room temperature (RT) for a period of 1 hour, followed by three rinses with TBST, each lasting 10 minutes. Subsequently, they were incubated with anti-USP14 (1:1000, ab192618, Abcam), anti-Bax (1:1000, ab32503, Abcam), Bcl-2 (ab185002, 1:1000, Abcam), CyclinD1 (1:1000, ab16663, Abcam), anti-Vimentin (1:1000, ab92547, Abcam), anti-Slug (1:1000, ab27568, Abcam), anti-E-cadherin (1:1000, ab40772, Abcam), anti-ZEB1 (1:1000, ab203829, Abcam), anti-snial (1:1000, ab216347, Abcam), anti-I-κB (1:1000, ab247825, Abcam), anti-NF-κB (1:1000, ab32536, Abcam), anti-p-NF-κB (1:1000, ab76302, Abcam), and anti-β-actin (1:1000, ab8227, Abcam) at 4°C overnight. Following the rinsing process with TBST, the membranes were incubated with horseradish peroxidase (HRP)-labeled secondary antibodies obtained from Abcam (ab6721) at a concentration of 1:1000 for 1 hour at RT. Subsequently, the membranes were subjected to three consecutive rinses with TBST, each lasting 10 minutes. Finally, WB special reagents from Invitrogen were utilized for color development and imaging, while ImageJ software was used to analyze the gray intensity.

### Dual-luciferase reporter assay

USP14-WT and USP14-MUT constructs were generated by Promega (Madison, WI, USA). For the experimental setup, EC cells at a seeding density of 4.5 × 10^4^ were plated in 48-well plates and cultured until reaching a fusion rate of 70%. Then, USP14-WT, USP14-MUT and miR-124-3p mimics or negative controls were cotransfected into endometrial cancer cells with Lipofectamine^®^ 3000 (Invitrogen; Thermo Fisher Scientific, Inc., Waltham, MA, USA). Luciferase activity was assessed following a 48-hour incubation period. The experimental procedure was repeated three times.

### Xenograft tumor experiment

A total of twenty BALB/c-nu nude mice, aged 6 weeks and weighing between 12–15 g, were acquired from the Animal Experimental Center of Tianjin Medical University. All mice were maintained under sterile conditions throughout the experiment. HEC-1-B cells were transfected with USP14 overexpression plasmids or negative vectors. The HEC-1-B cells (approximately 1 × 10^8^ cells/ml, 0.1 mL in volume) were gathered and subsequently administered via subcutaneous injection into the left forelimb of the mice. Following inoculation, the mice were provided with nourishment, and their overall states were diligently monitored. The appearance of a military nodule indicated successful modeling. On a weekly basis, the longitudinal and latitudinal dimensions of the nodules were assessed to compute the tumor volume. Upon completion of a five-week duration, the nude mice were euthanized. The subcutaneous nodules were excised, and subsequent calculations were performed to determine the tumor volume. The volume (V) was calculated according to the formula V=1/πab^2^ (a is the maximum diameter, and b is the minimum diameter of the tumor). The implementation of this animal experiment was duly approved and conducted under the auspices of the Ethics Committee of Tianjin Hospital. Careful considerations were taken to ensure that the tumor burden did not surpass the permissible dimensions.

### Immunohistochemistry

The tumor tissues obtained from the nude mice were meticulously preserved by fixation with 4% PFA (P0099, Beyotime, Shanghai, China) and subsequent embedding in paraffin. To facilitate further analysis, the tumor tissues were carefully sectioned into slices measuring 4 μm in thickness. These sections were subjected to a temperature of 65°C for 1 hour, followed by deparaffinization using xylene for two consecutive periods of 20 minutes each. Sequential immersion in ethyl alcohol with varying concentrations of 100%, 95%, 90%, 80%, and 70% was then carried out for 5 minutes each. To restore antigenicity, a process known as antigen retrieval was conducted by subjecting the sections to a temperature of 120°C in citrate buffer (pH 6.0) for 5 minutes. Next, the sections were incubated in 3% hydrogen peroxide after being washed with PBS twice. Bovine serum albumin (5%) (ST023, Beyotime, Shanghai, China) was used to block the sections for 1 h, followed by incubation with primary antibodies, including anti-Ki67 antibody (ab15580, 1:1000, Abcam) or anti-USP14/TGT antibody (ab235960, 1:250, Abcam), overnight at 4°C. After being washed with PBST three times, 50 μl of goat anti-rabbit IgG H&L (HRP) (ab205718, Abcam) was added to the slices, and the sections were incubated at RT for 1 h. For the development of color as a chromogen, 3′3-diaminobenzidine tetrahydrochloride was employed, while hematoxylin was utilized as a counterstain for a duration of 10 minutes to enhance the visual contrast of the slices. Ethanol and xylene at different concentrations were utilized to dehydrate the sections, and neutral balsam was applied to seal the sections. Finally, a microscope (Olympus Bx51, Japan, Tokyo) was used to photograph the sections by microscopy (Japan).

### Analysis of statistics

The statistical software package SPSS17.0 (SPSS Inc., Chicago, IL, USA) was employed for data analysis. The data are expressed as the mean ± standard deviation (x ± sd). To analyze differences among multiple groups, one-way ANOVA was employed, while for pairwise comparisons, the *t* test was utilized. Additionally, the chi-square analysis evaluated the variances in data presented in the fourfold table. When *P* < 0.05, statistical significance was corroborated. All statistical tests were conducted on a minimum of three occasions.

## RESULTS

### USP14 was overexpressed in EC and contributed to an unsatisfactory prognosis

**Figure 1:**
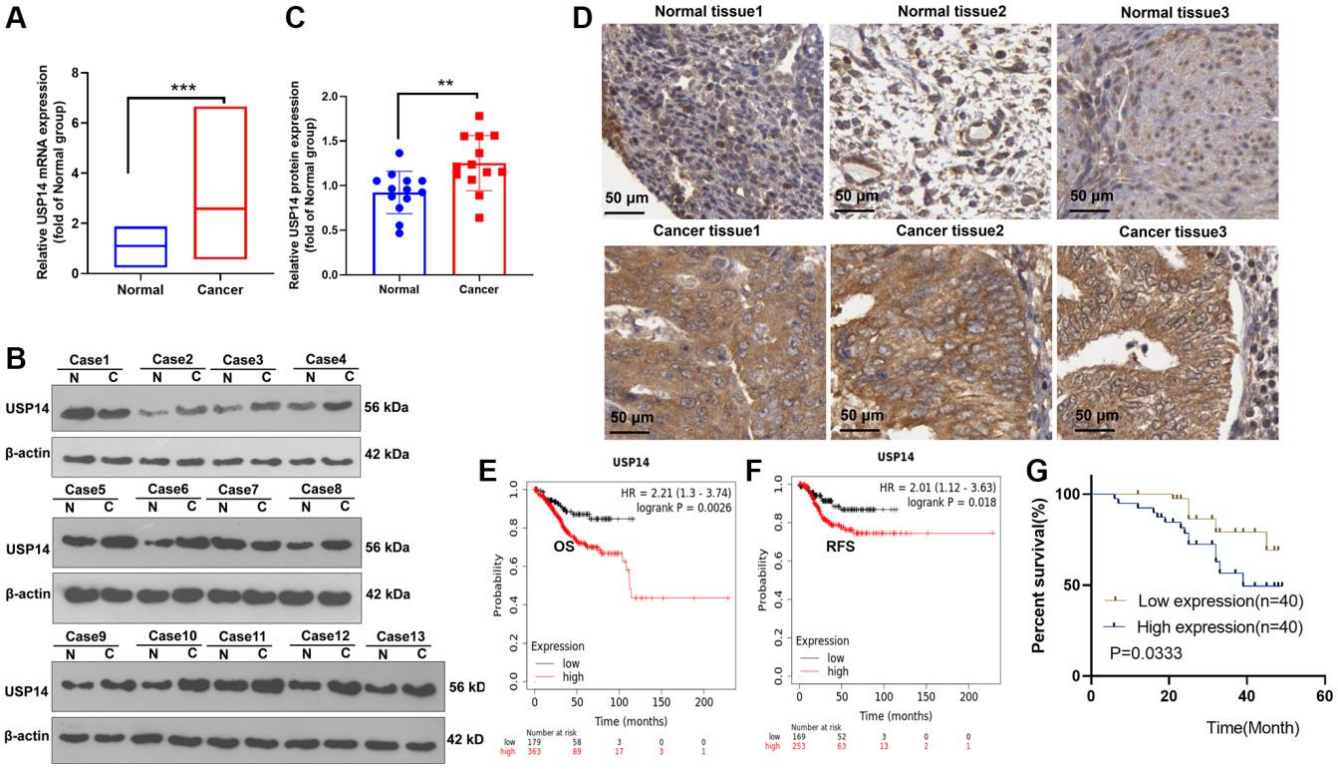
**Alteration of USP14 expression in EC.** (**A**–**C**) qRT-PCR (**A**) and WB (**B**, **C**) confirmed the USP14 level in EC tissues and paired adjacent normal tissues. (**D**) The Human Protein Atlas was used to analyze the USP14 profile in EC tissues and normal endometrial tissues. (**E**, **F**) The K-M plotter survival curve was adopted to clarify the correlation between USP14 and the overall survival (OS) and relapse-free survival (RFS) rates of EC patients. (**G**) The association between the USP14 level and the prognosis of EC patients was determined. ^**^refers to *P* < 0.01, ^***^represents *P* < 0.001. Abbreviations: N: Normal; C: endometrial carcinoma tissue.

We collected 44 EC tissues and their paired paracancerous normal tissues. qRT-PCR and WB techniques were employed to assess and track the expression levels of USP14 in the tissue samples. They were elevated in EC tissues (vs. paracancerous normal tissues) (Figure 1A–1C). Then, we analyzed the USP14 level in EC tissues and normal endometrial tissues through the Human Protein Atlas (https://www.proteinatlas.org/) to clarify the expression characteristics of USP14. USP14 was moderately or lowly expressed in normal endometrial tissues. In contrast, it was overexpressed in EC tissues, mainly in the cytoplasm and cell membranes (Figure 1D). Furthermore, the association between USP14 expression and EC prognosis was probed. Consequently, it was observed that patients exhibiting elevated levels of USP14 experienced a lower survival rate than those with lower USP14 levels (Figure 1E–1G). Moreover, we investigated the correlation between USP14 levels and the prognosis of EC. Intriguingly, it was found that patients with elevated USP14 levels exhibited greater tumor volume and an increased likelihood of lymph node metastasis and distant metastasis (Table 1). These findings suggested that USP14 was upregulated in EC and was implicated in distant metastasis and poor survival, indicating that USP14 contributed to EC.

**Table 1:** Table 1. Association between USP14 expression and clinicopathological characteristics in EC patients.

**Characteristics**	**Case**	**Expression of USP14**		***P* values**
		**Low**	**High**	
Age				
<60	20	9	11	0.824
≥60	24	10	14	
Clinical stage				
T1-T2	28	16	13	0.013^*^
T3-T4	16	3	12	
Differentiation				
Low	19	9	10	0.625
High	25	10	15	
Infiltration degree				
<1/2 Muscle layer	14	9	5	0.053
≥1/2 Muscle layer	30	10	20	
Lymph node metastasis				
Positive	17	3	14	0.0067^*^
Negative	27	16	11	
Distal metastasis				
Positive	14	10	4	0.0097^*^
Negative	30	9	21	

^*^Indicates *P* < 0.05.

### Overexpressing USP14 reduced apoptosis and promoted EC cell migration and invasion

**Figure 2:**
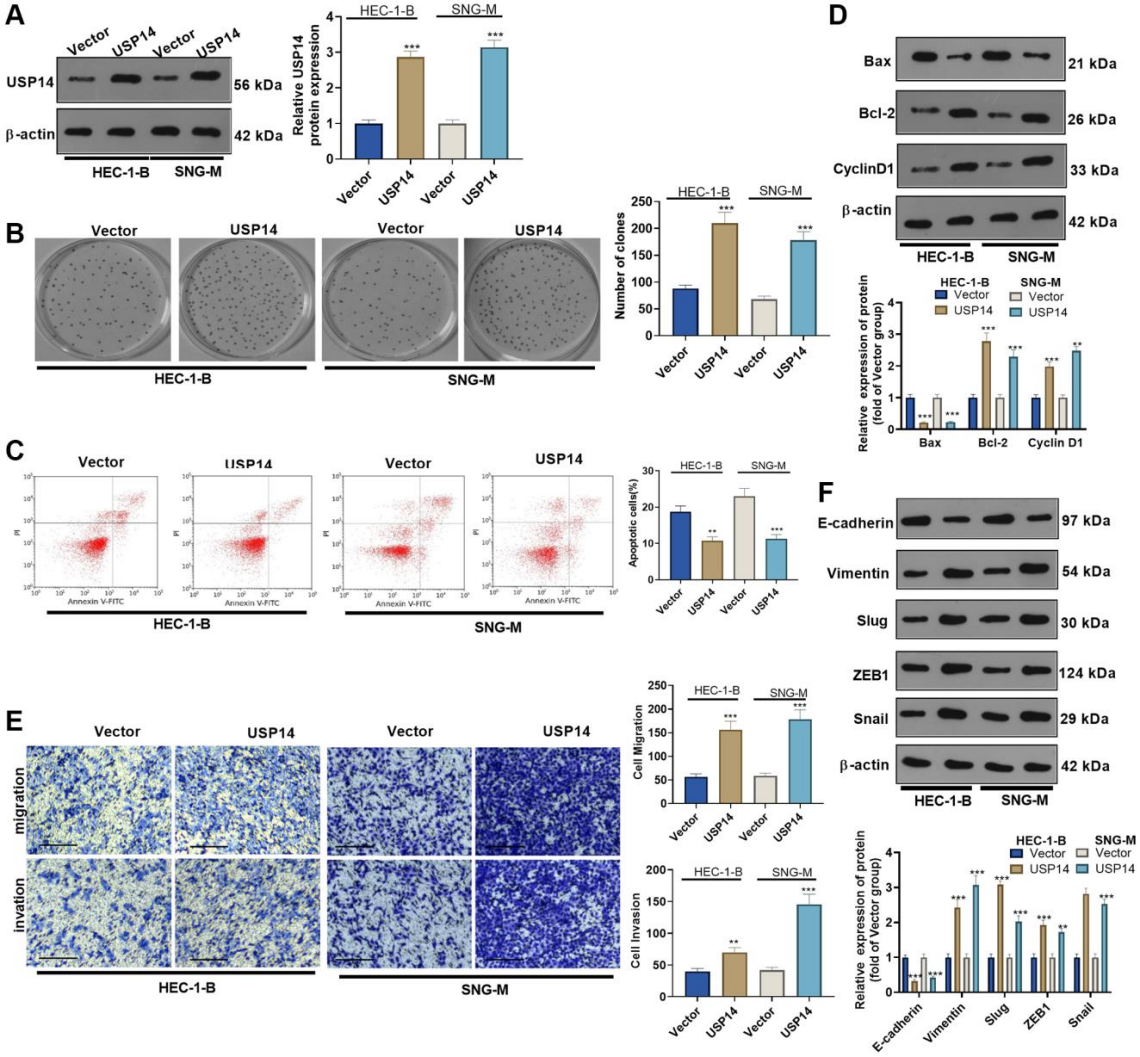
**The functions of USP14 in mediating the apoptosis, migration and invasion of EC cells.** (**A**) USP14 overexpression plasmids were transfected into HUC-1-B and SNG-M cells, and the USP14 profile in EC cell lines was examined via WB. (**B**) Colony formation of HEC-1-B and SNG-M cells was observed through colony formation experiments. (**C**) FCM verified apoptosis. (**D**) Bcl-2, Bax, and Cyclin D1 profiles were examined through WB. (**E**) Transwell assays were used to evaluate EC cell migration and invasion. (**F**) E-cadherin, Vimentin, Snail1, ZEB1 and Slug profiles were examined by WB. ^**^, ^***^represent *P* < 0.01, and *P* < 0.001, respectively. *N* = 3.

We transfected USP14 overexpression plasmids into HEC-1-B and SNG-M cells to clarify the specific mechanism of USP14 in EC. First, WB showed that USP14 was highly expressed in EC cells, indicating successful transfection (Figure 2A). The assessment of EC cell proliferation and apoptosis was performed using the colony formation assay and FCM, respectively. The results revealed that upregulating USP14 facilitated the colony-forming ability of HEC-1-B and SNG-M and reduced apoptosis (vs. the vector group, Figure 2B, 2C). Furthermore, apoptosis-related proteins (Bcl-2, Bax, and Cyclin D1) were compared by WB. As a result, overexpressing USP14 dampened the Bax level and enhanced the Bcl-2 and Cyclin D1 profiles (Figure 2D). Subsequently, the Transwell experiment assessed the impact of USP14 on EC cell migratory and invasive capabilities. Interestingly, cell migration and invasion were improved after USP14 overexpression (vs. the vector group) (Figure 2E). In addition, the profiles of EMT-related proteins (E-cadherin and Vimentin) and EMT-related transcription factors (Snail1, ZEB1, and Slug) were compared by WB. USP14 overexpression elevated the profiles of Vimentin, Snail1, ZEB1, and Slug while repressing the E-cadherin level (compared with the vector group, Figure 2F). These findings illustrated that overexpressing USP14 facilitated EC cell migration, invasion, and EMT.

### Overexpressing USP14 facilitated the growth of EC cells

**Figure 3:**
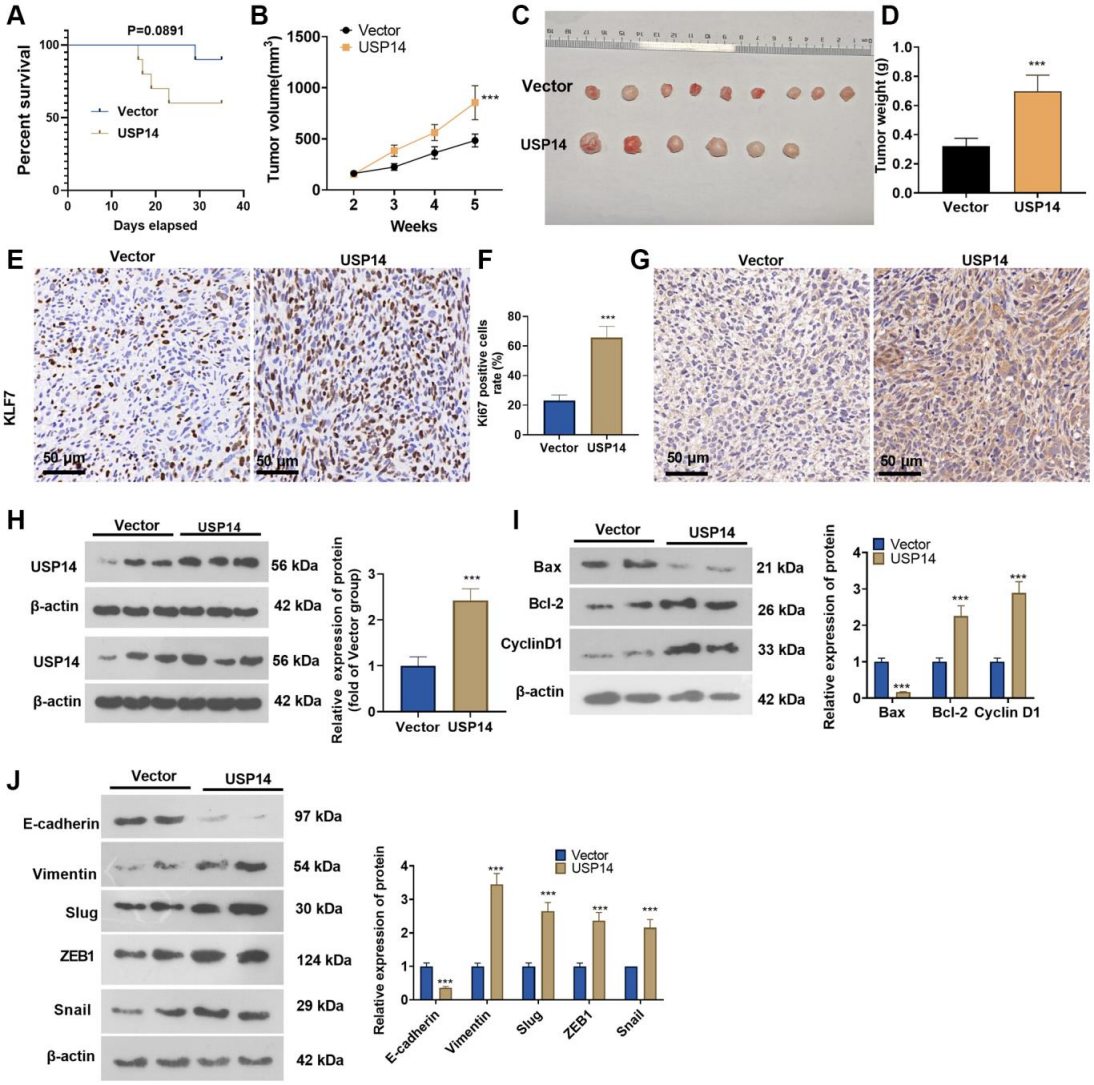
**Overexpressing USP14 increased EC cell growth *in vivo.*** USP14 overexpression plasmids were introduced into HEC-1-B cells through transfection, and a xenograft tumor model was established. (**A**) The survival rate of mice in the two groups is shown. (**B**) Tumor volumes in the nude mice. (**C**) Tumor images. (**D**) Tumor weight. (**E**, **F**) IHC was conducted to detect KI67 expression, and the KI67-positive cell rate was calculated. (**G**, **H**) IHC and WB were conducted to detect USP14 expression. (**I**, **J**) The apoptosis- and EMT-associated protein levels in tumor tissues were determined by WB. ^***^represents *P* < 0.001, compared to the vector group.

We introduced USP14 overexpression plasmids into HEC-1-B cells through transfection and built a xenograft model to corroborate the involvement of USP14 in the *in vivo* growth of EC. The survival analysis showed that the nude mice in the USP14 group had a lower survival rate (*p* = 0.0891) (Figure 3A). Overexpressing USP14 increased tumor volume and weight (Figure 3B–3D). We conducted IHC to detect cell proliferation *in vivo*. The results demonstrated that the USP14 group exhibited an elevated proportion of KI67-positive cells (Figure 3E, 3F). IHC and WB results revealed an upregulation of the USP14 protein level in the tumor tissues of the USP14 group compared to the vector group (Figure 3G, 3H). Next, we examined EMT- and apoptosis-related proteins. The results showed that Bcl-2, Cyclin D1, Vimentin, Snail1, ZEB1, and Slug were upregulated, while Bax and E-cadherin were downregulated in the USP14 group (Figure 3I, 3J). Thus, USP14 exerted an oncogenic effect on EC.

### Downregulating USP14 induced inhibitory effects in EC cells by promoting apoptosis

**Figure 4:**
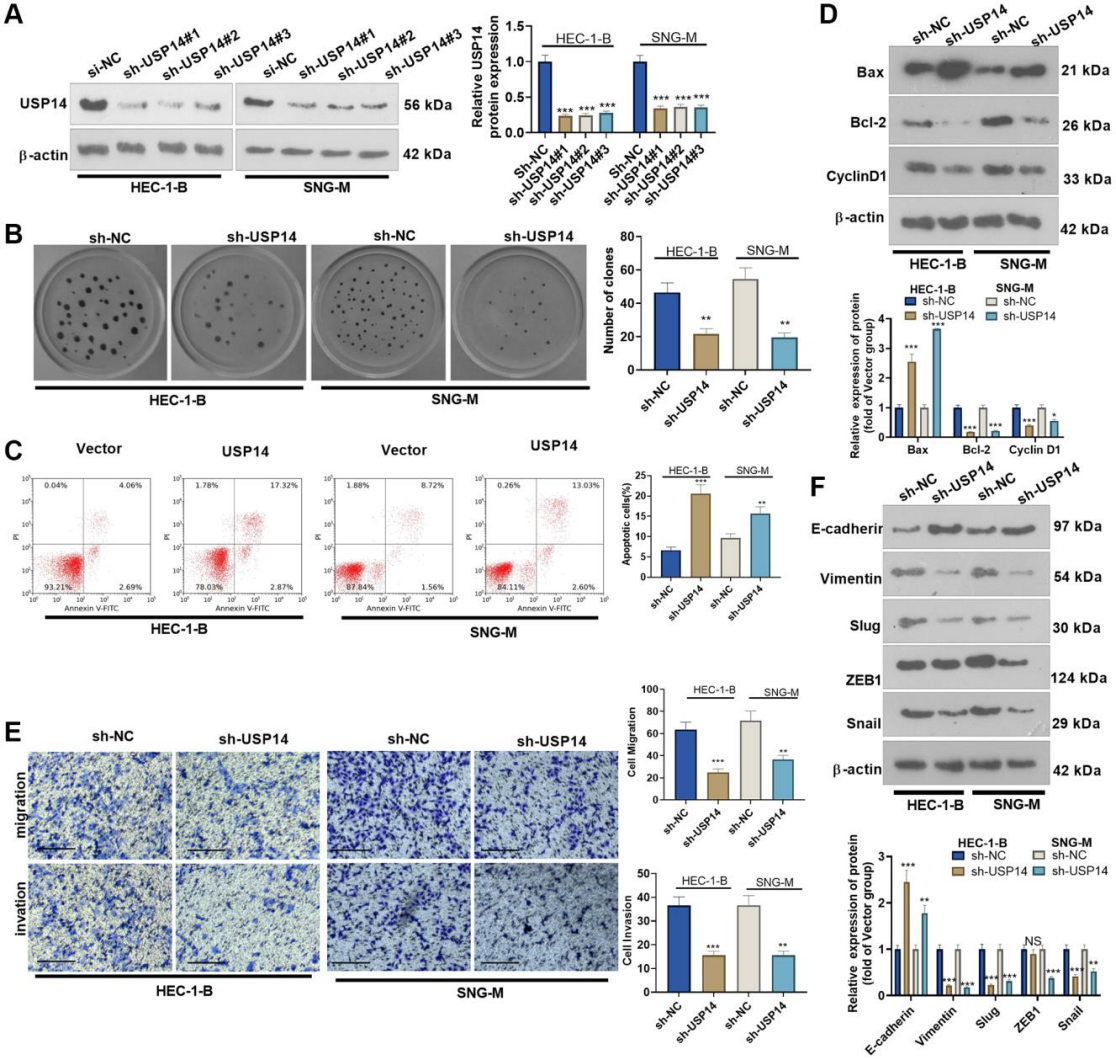
**The effects of USP14 knockdown in the apoptosis, migration and invasion of EC cells.** (**A**) sh-USP14 or sh-NC was transfected into HUC-1-B and SNG-M cells, and the USP14 profile in EC cell lines was examined by WB. (**B**) Colony formation of HEC-1-B and SNG-M cells was tracked by colony formation experiments. (**C**) FCM was used to observe apoptosis. (**D**) The expression levels of Bcl-2, Bax, and Cyclin D1 were examined by WB. (**E**) Transwell assays were implemented to evaluate EC cell migration and invasion. (**F**) E-cadherin, Vimentin, Snail1, ZEB1, and Slug profiles were examined by WB. NS, ^*^, ^**^, ^***^indicate *P* > 0.05, *P* < 0.05, *P* < 0.01, and *P* < 0.001, respectively, vs. sh-NC. *N* = 3.

We transfected sh-USP14 into HEC-1-B and SNG-M cells. The transfection efficiency was confirmed by WB (Figure 4A). Colony formation experiments and FCM were conducted to determine EC cell proliferation and apoptosis, respectively. The results revealed that USP14 knockdown repressed the colony-forming ability of HEC-1-B and SNG-M cells and enhanced apoptosis (vs. the vector group, Figure 4B, 4C). WB results indicated that sh-USP14 enhanced the Bax level and reduced Bcl-2 and Cyclin D1 expression (Figure 4D). By detecting cell migration and invasion by Transwell assay, it was found that USP14 knockdown reduced EC cell migration and invasion (Figure 4E). Furthermore, WB results revealed that USP14 knockdown enhanced E-cadherin levels while reducing Vimentin, Snail1, ZEB1, and Slug profiles (Figure 4F). As a result, USP14 knockdown exerted antitumor functions in EC cells.

### miR-124-3p targeted USP14 and inhibited its expression

**Figure 5:**
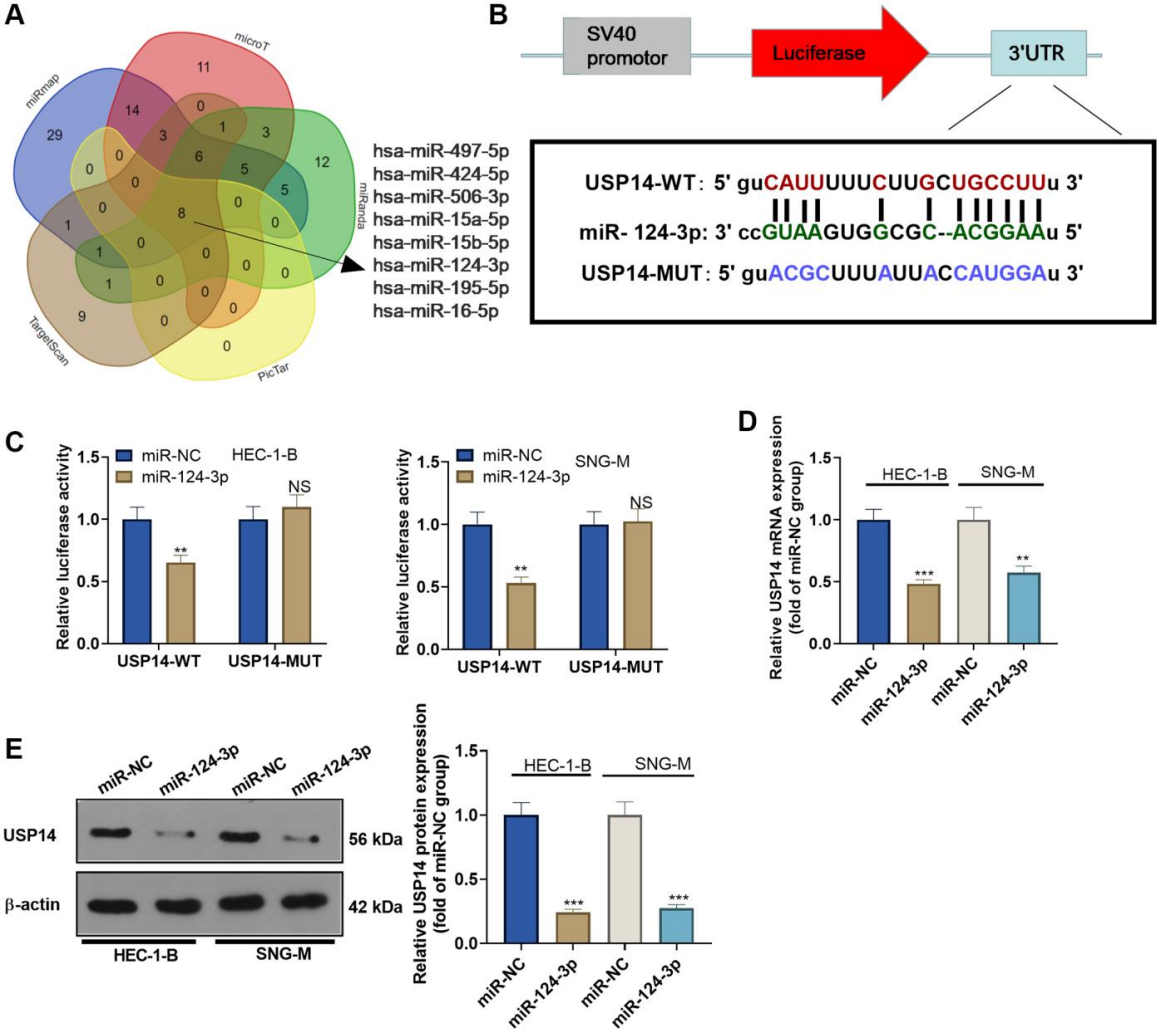
**miR-124-3p targeted and repressed USP14.** (**A**) StarBase was employed to analyze the upstream miRNAs of USP14, and a Venn diagram was adopted to analyze the USP14-targeted miRNAs shared by miRmap, microT, miRanda, PicTar, and TargetScan. (**B**) The binding sites between miR-124-3p and USP14. (**C**) The targeting correlation between miR-124-3p and USP14 was verified by dual-luciferase reporter assay. (**D**, **E**) miR-124-3p mimics were transfected into EC cells, and USP14 mRNA and protein expression was assessed. NS, ^**^, ^***^represent *P* > 0.05, *P* < 0.01, and *P* < 0.001. *N* = 3.

Given the established pro-oncogenic role of USP14 in EC, our curiosity was piqued regarding its upstream regulatory pathway. By consulting the Starbase (http://starbase.sysu.edu.cn/index.php), we discovered that hsa-miR-497-5p, hsa-miR-424-5p, hsa-miR-506-3p, hsa-miR-15a-5p, hsa-miR-15b-5p, hsa-miR-124-3p, hsa-miR-195-5p, and hsa-miR-16-5p are potential target genes of USP14 (Figure 5A). Among them, miR-124-3p has been reported as a target of USP14 [13]. Thus, to validate the targeting correlation between the two entities, we employed the dual-luciferase reporter assay. Consequently, our findings revealed that the introduction of miR-124-3p mimics diminished the luciferase activity specifically in USP14-WT-transfected EC cells, while it exhibited no impact on USP14-MUT-transfected EC cells (Figure 5B, 5C). Subsequently, we introduced miR-124-3p mimics into EC cells and observed a reduction in USP14 expression upon overexpression of miR-124-3p (Figure 5D, 5E), illustrating that miR-124-3p was the upstream regulatory molecule of USP14 and negatively regulated USP14.

### Overexpressing miR-124-3p reversed the USP14-mediated oncogenic effects

**Figure 6:**
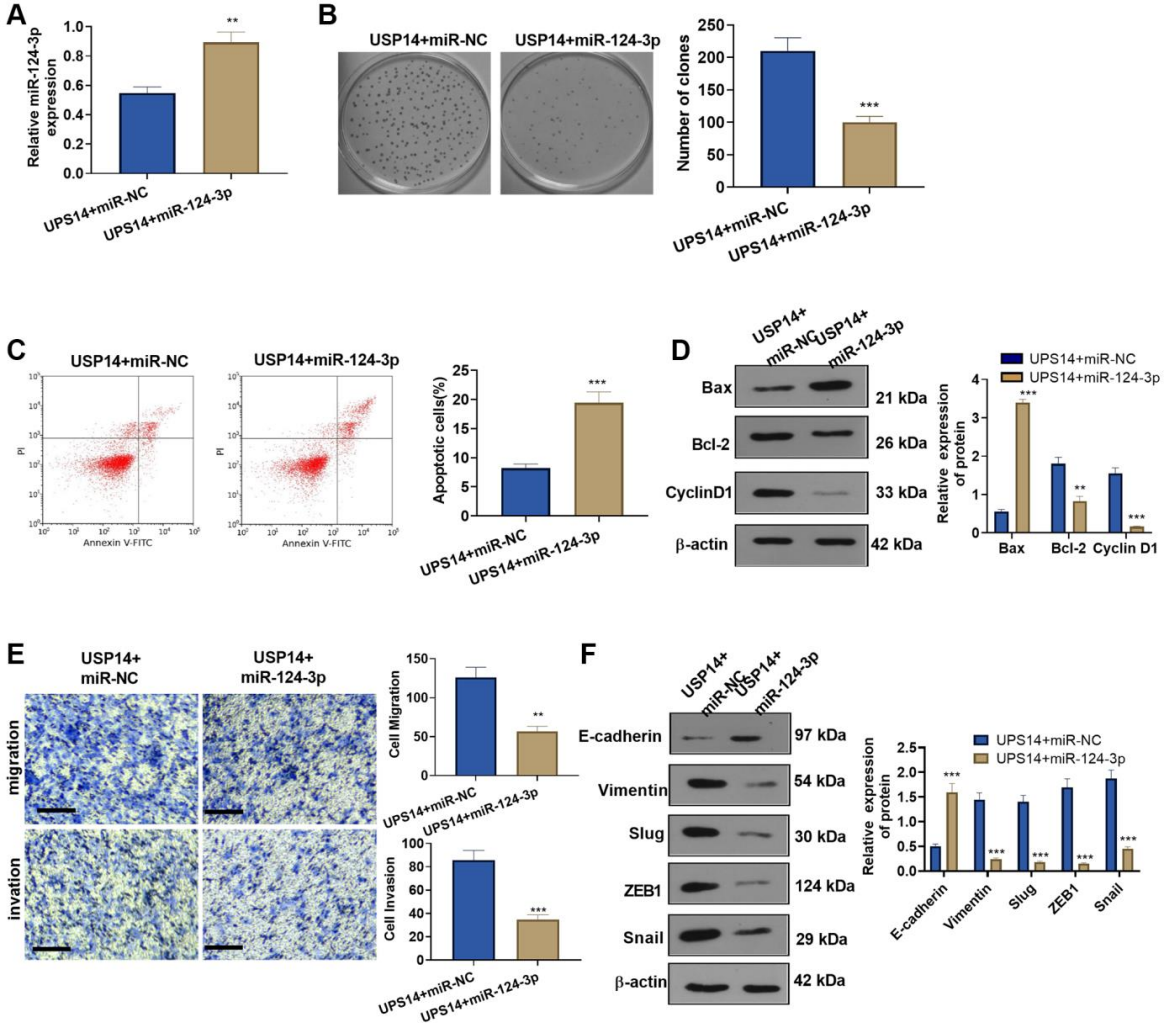
**Overexpressing miR-124-3p reversed USP14-mediated oncogenic function.** (**A**) miR-124-3p mimics or miR-NC were transfected into USP14-overexpressing HEC-1-B cells, and qRT-PCR was used to check the miR-124-3p profile. (**B**) The colony formation experiment verified the colony formation of HEC-1-B and SNG-M cells. (**C**) FCM was implemented to verify EC apoptosis. (**D**) Bcl-2, Bax, and Cyclin D1 protein profiles were compared by WB. (**E**) Transwell assays were used to test EC cell migration and invasion. (**F**) The levels of E-cadherin, Vimentin, Snail1, ZEB1, and Slug were tested by WB. ^**^, ^***^indicate *P* < 0.01 and *P* < 0.001, vs. USP14+miR-NC. *N* = 3.

To gain further insights into the miR-124-3p-USP14 axis in EC cells, we transfected miR-124-3p mimics into USP14-overexpressing cells (Figure 6A). Subsequently, detection of EC cell proliferation and apoptosis was carried out. Our findings showed that vis-à-vis the USP14+miR-NC group, the colony formation of EC cells in the USP14+miR-124-3p group was weakened (Figure 6B), and apoptosis was increased (Figure 6C). In addition, Bax levels increased in the USP14+ miR-124-3p group, while Bcl-2 and Cyclin D1 levels were decreased (vs. USP14+miR-NC group, Figure 6D). Furthermore, EC cell migration, invasion, and EMT were examined, and it was found that after miR-124-3p upregulation, the migration and invasion of USP14-overexpressing EC cells were reduced (Figure 6E). Meanwhile, Snail1, ZEB1, and Slug were downregulated, whereas E-cadherin was elevated (Figure 6F). Taken together, these observations provided compelling evidence demonstrating the ability of miR-124-3p to counteract the oncogenic impact of USP14.

### USP14 initiated the NF-κB signaling pathway and mediated EMT

**Figure 7:**
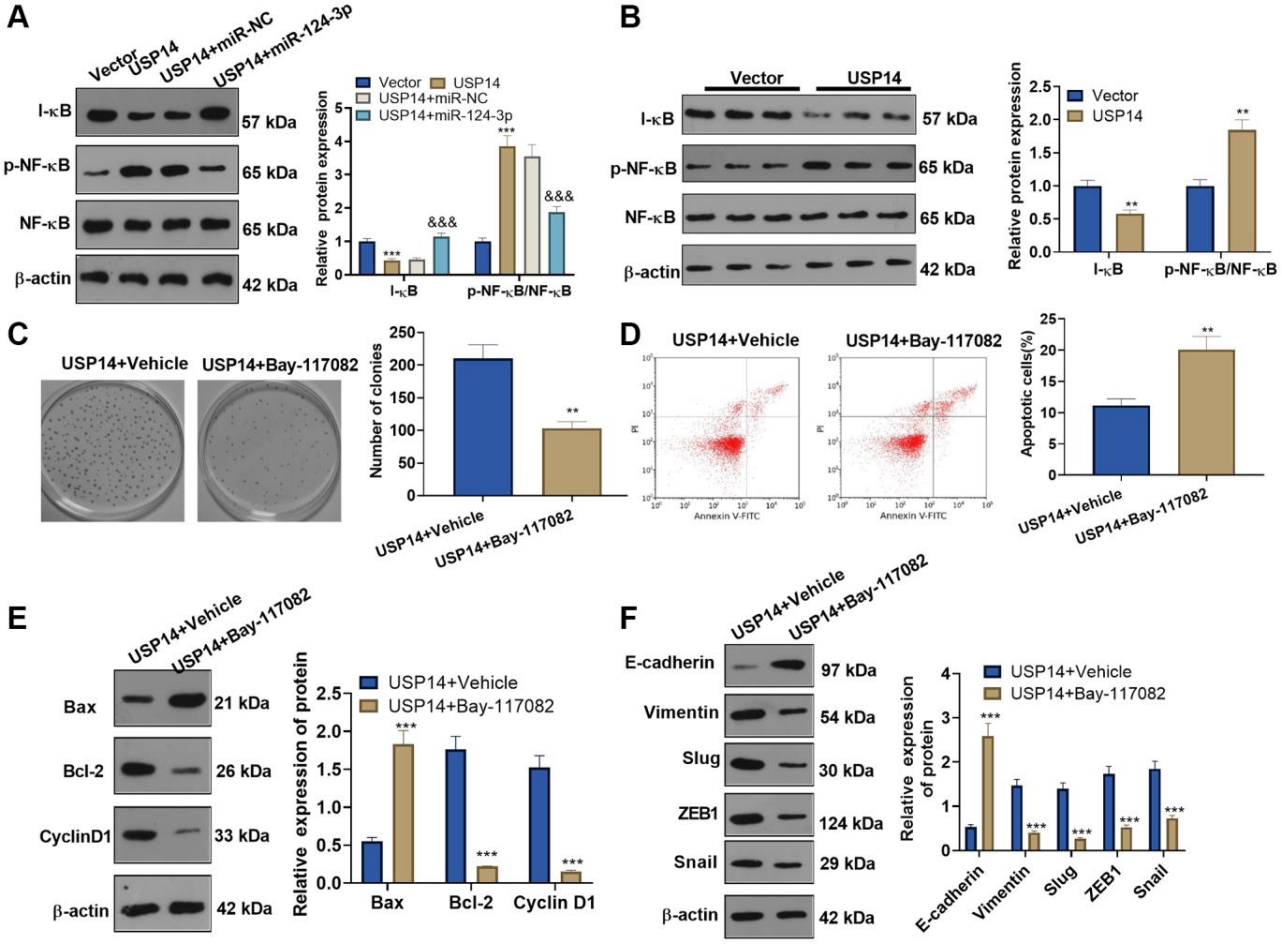
**USP14 activated the NF-κB pathway and mediated EMT.** (**A**) USP14 overexpression plasmids and/or miR-124-3p mimics were transfected into HEC-1-B cells, and the profiles of I-κB and NF-κB in EC cells were examined by WB. ^**^ and ^***^represent *P* < 0.01 and *P* < 0.001, (vs. vector). ^&&&^indicates *P* < 0.001 (vs. USP14+miR-NC). (**B**) The levels of I-κB and NF-κB in tumors were detected by WB, ^**^indicates *P* < 0.01, vs. Vector. (**C**) USP14-overexpressing cells were treated with BAY-1170829 (2 μM) to test the colony formation of HEC-1-B cells. (**D**) FCM was used to track EC apoptosis. (**E**) The protein profiles of Bcl-2, Bax, and Cyclin D1 were monitored by WB. (**F**) The levels of E-cadherin, Vimentin, Snail1, ZEB1, and Slug were compared by WB. ^**^ and ^***^represent *P* < 0.01 and *P* < 0.001, respectively (vs. USP14+vehicle). *N* = 3.

**Figure 8:**
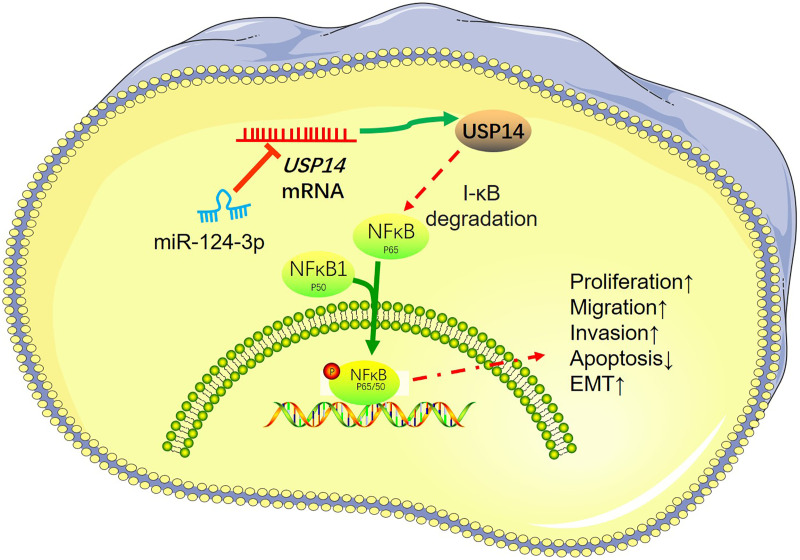
A mechanism scheme of USP14 in endometrial carcinoma.

WB was conducted to verify I-κB and NF-κB expression in EC cells. The results revealed that USP14 upregulation reduced the I-κB level and elevated NF-κB phosphorylation (Figure 7A). Conversely, following the introduction of miR-124-3p mimics, the expression of I-κB was observed to be enhanced, accompanied by a reduction in NF-κB phosphorylation (Figure 7A). In tumor tissues, USP14 upregulation dampened I-κB and strengthened NF-κB phosphorylation (Figure 7B). To gain additional elucidation regarding the mechanism of the NF-κB pathway in USP14-mediated oncogenic effects, USP14-overexpressing cells were transfected with the NF-κB inhibitor Bay-117082. The outcomes demonstrated that in contrast to the USP14 group, cell proliferation in the USP14+ Bay-117082 group was reduced (Figure 7C), apoptosis was increased (Figure 7D), Bcl-2 and CyclinD1 were downregulated, and Bax was upregulated (Figure 7E). Moreover, we discovered that EC cell EMT was repressed after Bay-117082 treatment (Figure 7F). In summary, these results substantiated that USP14 initiated the NF-κB signaling pathway to induce EMT and suppress apoptosis (Figure 8).

## DISCUSSION

EC is the most common malignancy in the female reproductive tract in developed countries, which has an incidence of 5.9% [14, 15]. Based on the presence of clinicopathological risk factors, adjuvant treatment can improve both recurrence-free and overall survival of EC patients [16]. Thus, investigating novel targets of EC helps the diagnosis and targeted therapy. Here, it was ascertained that the USP14 profile was heightened in both EC tissues and cells. Notably, this upregulation of USP14 was found to enhance the migratory and invasive capabilities of EC cells (Figure 8).

The ubiquitin proteasome system (UPS) stands as the primary regulatory apparatus governing protein degradation, exerting its dominance over fundamental cellular mechanisms. DUB is upregulated in diverse human cancers, and its abnormal activity pertains to cancer progression and the onset of chemical resistance [17, 18]. Previous studies have shown that USP14 profiles are implicated in ovarian cancer [19], lung adenocarcinoma [20], non-small cell lung cancer [21] and neuroblastoma [22]. Additionally, USP14 is an underlying biomarker for EC diagnosis and a molecular target for EC treatment [23]. In this work, we verified the overexpression of USP14 in both EC cell lines and tissues, unraveling its propensity to enhance the migratory and invasive capabilities of EC cells. Additionally, USP14 was found to dampen apoptosis and stimulate the EMT process in EC cells.

Immunotherapeutic approaches have been regarded as a promising strategy for EC treatment whether by single immune checkpoint inhibitors or combining with traditional chemotherapeutic drugs [24]. Poly (ADP-ribose) polymerase (PARP) inhibitors and PD-1/PD-L1 checkpoint inhibitors are two major immune therapies, and they achieve a synergistic effect against EC [25]. Interestingly, USP14 plays a role in the immune therapy of cancers. For example, USP14 has enhanced expression in head and neck squamous cell carcinomas and promotes TNF-α-inducible NF-κB activity. It should be noted that TNF-α can activate the canonical NF-κB pro-survival pathway in cancers, thus contributing to therapy resistance [26]. In another study, USP14 downregulation mitigates the K63-linked IRF3 deubiquitylation. Downregulating USP14 increases dendritic cells (DCs) sensing of irradiated-tumor cells *in vitro*, suggesting that repressing USP14 promotes systemic antitumor immunity [27]. Therefore, USP14 might also be a potential immunotherapeutic target in EC.

The NF-κB factor, an essential transcription factor located in the nucleus, is made up of the NF-κB family along with its inhibitor, the IκB family [28]. It exists in a variety of cells and is involved in inflammation, immunity, cell proliferation, apoptosis, etc., [29, 30]. NF-κB activation is an essential mechanism of tumor cell resistance to antitumor drugs such as TNF [31]. Inactivating NF-κB by inhibiting IκB degradation leads to a large amount of tumor cell apoptosis [32]. Additionally, NF-κB upregulates multiple transcription factors, such as Slug, ZEB1, and Snail1, which directly enhance tumor cell EMT, invasion, and migration [33]. In addition, abnormal activation of NF-κB is a critical factor leading to the malignant progression of EC [34]. Therefore, inhibiting NF-κB activity through gene therapy, combined with routine chemotherapy, is expected to be an effective tumor therapy. Interestingly, USP14 overexpression has been shown to be closely related to NF-κB activation [11, 12]. In this study, USP14 overexpression inhibited I-κB and increased NF-κB phosphorylation, accompanied by increased EC cell migration, invasion, and EMT. After intervention with NF-κB inhibitors, the carcinogenic effect mediated by USP14 overexpression was weakened. Thus, USP14 promotes EC progression in an NF-κB pathway-dependent manner.

Several miRNAs have been identified to target and regulate USP14, thus affecting tumor cells. For example, the downregulation of miR-320a increases the stability of vimentin in gastric cancer (GC) cells by enhancing USP14, thus upregulating vimentin and promoting GC cell growth, migration, and invasion [35]. miR-4782-3p impedes cell proliferation in non-small cell lung cancer (NSCLC) by targeting USP14, ZEB2, and XIAP [36]. Here, we searched online websites and discovered that miR-124-3p targeted USP14. Moreover, the dual-luciferase reporter gene experiment indicated that the two are negatively correlated. Furthermore, overexpressing miR-124-3p dampened USP14 in EC and reversed the carcinogenic effect of USP14.

At the posttranscriptional level, the modulation of genes occurs through the attenuation of messenger RNA (mRNA) translation or the promotion of mRNA degradation, facilitated by small noncoding RNAs known as miRNAs [37]. miRNA expression disorders have been found in almost all human cancers, and many miRNAs contribute to tumorigenesis and tumor development by regulating oncogenes and tumor suppressor genes [38]. miR-124-3p has been confirmed to contribute to GC [39], bladder cancer [40], breast cancer [41], and glioma [42]. Additionally, the abnormal profiles of miRNAs affect EC progression. For example, miR-522 accelerates EC cell proliferation, migration, and invasion by inhibiting monoamine oxidase B (MAOB) [43]. miR-29a-5p downregulates EC-derived cell proliferation and invasion and enhances apoptosis through TPX2 [44]. Nevertheless, the mechanism of miR-124-3p in EC remains elusive. We discovered that miR-124-3p was repressed in EC cells, and its overexpression reversed the carcinogenic effect mediated by USP14, reduced cell proliferation, invasion, and migration, and increased cell death. These findings verified the tumor-suppressive effect of miR-124-3p in EC.

There are several limitations that should be considered in our future study. First, there might be other miRNAs that can also affect USP14 expression and EC development. The other miRNAs that regulate USP14 in EC need further exploration. Second, more clinical samples of EC should be collected to confirm the predictive role of miR-124-3p-USP14 axis in EC diagnosis. Third, we performed *in vivo* assays and confirmed that USP14 promoted EC cell growth. The functions of the miR-124-3p-USP14 axis in EC growth need to be confirmed in animal studies.

## CONCLUSION

The present investigation reveals that miR-124-3p-mediated modulation of USP14 exacerbates the progression of EC through NF-κB pathway activation. Manipulation of this pathway represents a novel and promising therapeutic approach for the management of EC. This study provides a deeper understanding of the intricate molecular mechanisms underlying EC and offers valuable insights for the development of effective treatment protocols for this disease.
